# Genetic polymorphism, constitutive expression and tissue localization of *Dirofilaria immitis* P-glycoprotein 11: a putative marker of macrocyclic lactone resistance

**DOI:** 10.1186/s13071-022-05571-6

**Published:** 2022-12-21

**Authors:** Emily Curry, Roger Prichard, Anne Lespine

**Affiliations:** 1 Institute of Parasitology, McGill University, Sainte Anne-de-Bellevue, Montreal, QC Canada; 2 INTHERES, INRAE, ENVT, Université de Toulouse, 31027 Toulouse Cedex 3, France

**Keywords:** *Dirofilaria immitis*, Dirofilariosis, Macrocyclic lactones, Resistance, P-glycoprotein 11, MiSeq sequencing, Constitutive expression, Immunofluorescence assay

## Abstract

**Background:**

*Dirofilaria immitis* causes dirofilariosis, a potentially fatal condition in canids. *Dirofilaria* infections can be prevented with a macrocyclic lactone (ML) prophylactic regimen. However, some *D. immitis* isolates have become resistant to MLs. Genetic changes on the P-glycoprotein 11 gene, encoding an ABCB transporter, have been linked to the ML-resistant phenotypes and have been proposed as markers of drug resistance. However, nothing is known about the expression and the localization of this transporter in *D. immitis*, despite its strong link to ML-resistant phenotypes.

**Methods:**

We examined the clinically validated *D. immitis* P-glycoprotein 11 (*Dim*Pgp-11) single nucleotide polymorphism (SNP) via MiSeq analysis in three ML-susceptible isolates (Missouri, MP3 and Yazoo) and two ML-resistant isolates (JYD-34 and Metairie), and correlated the data with previously published MiSeq results of USA laboratory-maintained *D. immitis* isolates. The level of the expression of the *Dim*Pgp-11 messenger RNA transcript was analyzed by droplet digital PCR (ddPCR) and compared in the USA laboratory-maintained isolates, namely the ML-susceptible Missouri and Berkeley isolates, the putative ML-susceptible Georgia III and Big Head isolates and the ML-resistant isolate JYD-34. The immunolocalization of *Dim*Pgp-11 was visualized in the microfilaria (mf) life stage of the Missouri isolate using confocal microscopy.

**Results:**

The results confirmed that the SNP found on *Dim*Pgp-11 is differentially expressed in the USA laboratory-maintained isolates. The ML-susceptible isolates had an alternate allele frequency of between 0% and 15%, while it ranged between 17% and 56% in the ML-resistant isolates. The constitutive expression of *Dim*Pgp-11 was similar in the Berkeley, Georgia III and Big Head isolates, while it was significantly decreased in the ML-resistant JYD-34 isolate (*P* < 0.05), when compared to the ML-susceptible Missouri isolate. The *Dim*Pgp-11 protein was distinctly localized within the excretory-secretory (ES) duct, pore cells and the excretory cell and, more faintly, along the mf body wall.

**Conclusion:**

Our data confirm that genetic polymorphism of *Dim*Pgp-11 is associated with ML resistance in USA laboratory-maintained *D. imminits* isolates. A link between *Dim*Pgp-11 and ML resistance in *D. immitis* is further supported by the lower protein expression in the ML-resistant JYD-34 isolate when compared with the ML-susceptible Missouri isolate. Interestingly, *Dim*Pgp-11 is strategically located surrounding the ES pore where it could play an active role in ML efflux.

**Graphical Abstract:**

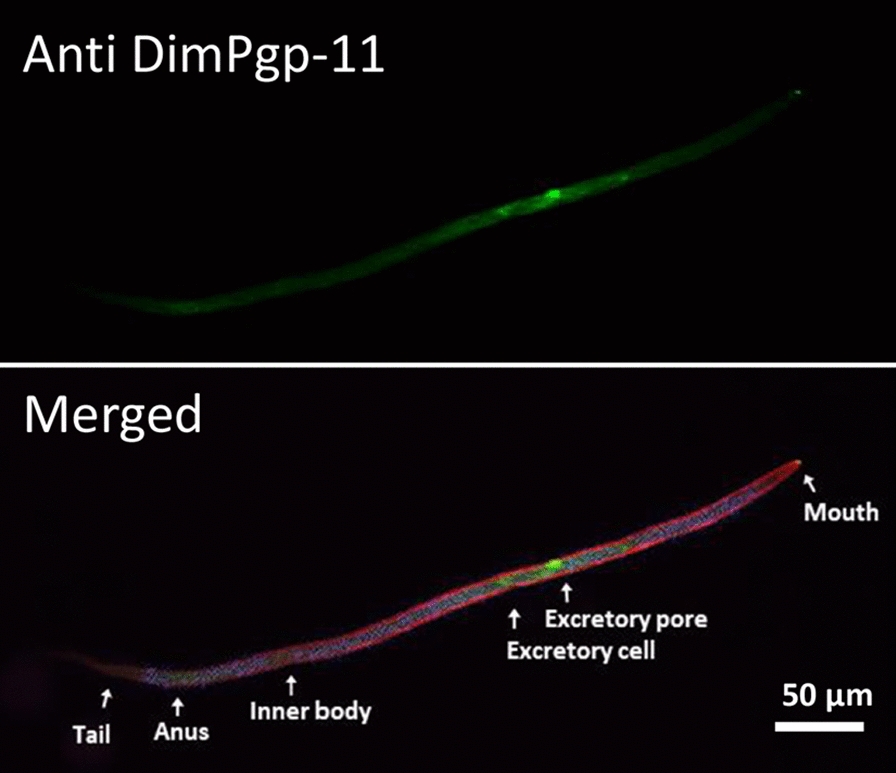

**Supplementary Information:**

The online version contains supplementary material available at 10.1186/s13071-022-05571-6.

## Background

The parasitic nematode *Dirofilaria immitis* is the causative agent of dirofilariosis, a pulmonary infection which primarily affects canids and felines. The macrocyclic lactones (MLs) remain the only class of drug approved for prophylactic treatment to prevent the development of dirofilariosis infection [1–4]. In *D. immitis*, the MLs act against larval stages, killing the developing third- and fourth-stage (L3/L4) larvae and preventing the establishment of adult infection, which causes the pathology. They are used at low dose rates; for example 6–12 µg/kg body weight for ivermectin (IVM) [5]. At slightly higher dose rates the MLs are also microfilaricidal and reduce the fecundity of adult parasites [5]. MLs are generally safe drugs for preventing *Dirofilaria* infections and have been used for dirofilariosis prevention since 1987.

Heartworm chemoprophylactives are required to be 100% effective at preventing the establishment of *D. immitis* infections in registration trials [5]. Thus, in theory, any decrease in efficacy below 100% could be considered as resistance. The establishment of *D. immitis* infections in animals on ML-based heartworm preventatives indicates a loss of efficacy of the drug and a potentially ML-resistant population. However, the establishment of an infection does not itself prove ML resistance in *D. immitis*. A missed treatment or a delay in the scheduled treatment may be the cause of the establishment of infection, unrelated to resistance. It is difficult to prove unequivocally that heartworm prevention has been wholly in compliance with the recommended treatment. Furthermore, a resistance phenotype may depend on the dose rate, the number of treatments administered, the formulation and the MLs administered [5]. As true drug resistance is genetic, it is heritable [6]. Since resistance to ML in nematodes is a complex mechanism that may involve multiple genes, the conditions for unequivocally determining ML resistance can be quite challenging.

The P-glycoprotein (Pgp) efflux transporters have been identified as one of the relevant factors affecting ML efficacy and contributing to ML resistance in nematodes. Pgps belong to a class of protein known for their involvement in ML pharmacology as efflux pumps [7–13]. MLs bind with high affinity on the Pgps in the hydrophobic binding pocket, the first of a series of events, including ATP hydrolysis, that furnish the energy needed for drug translocation out of the cells and the organisms [11]. Changes on Pgps, genetic or induced, may impact the pump activity, subsequently leading to lower drug efficiency.

Genetic changes associated with the ML loss of efficacy (LOE) phenotype were documented in a subset of genes in 2011, including the gene for a yet unidentified Pgp [14, 15]. Two single nucleotide polymorphisms (SNPs) in the Pgp gene, at position 11 and 618, respectively, were found to be highly associated with the LOE phenotype. One of these, the SNP A11G, is located in a coding region and confers an amino acid change from lysine to arginine at position 1203 within the second nucleotide binding domain (NBD) adjacent to the Walker B site [16, 17]. This change introduces a basic polar group prior to the ATP binding domain, and similar changes at this position have been shown to modulate ATP turnover and the transport activity of the mammalian transporter homolog [18]. The second SNP, A618G, is located in a non-coding region. These two SNPs affecting the *D. immitis* Pgp were combined for diplotypic analysis and are known as the GG-GG genotype. Whole genome-wide association determined that *D. immitis* possesses three functional full-length Pgps: P-glycoprotein 3 (*Dim*Pgp-3), P-glycoprotein 10 (*Dim*Pgp-10) and P-glycoprotein 11 (*Dim*Pgp-11) [16]. The diplotypic GG-GG genotype was isolated on *Dim*Pgp-11 [19]. The *Dim*Pgp-11 diplotypic GG-GG genotype has been used as one of four criteria to help validate the heritability of true ML resistance in *D. immitis* [6].

The SNP A11G is the focus of the research reported here and is hereafter referred to as the *Dim*Pgp-11 SNP. The Pgp-11 gene has been associated with the development of ML resistance in the model organism *Caenorhabditis elegans* and several nematode species of veterinary importance, making *Dim*Pgp-11 a gene of interest for characterization in the context of ML resistance. Pgp-11 in *C. elegans *(*Cel*Pgp-11) is strategically located within the intestine and the excretory cell [20–22]. ML-resistant *C. elegans* isolates have been shown to constitutively overexpress several genes involved in xenobiotic metabolism and transport, including *Cel*Pgp-11 [23, 24]. The parasitic intestinal nematode of cattle, *Cooperia oncophora*, shows a three- to fivefold upregulation of P-glycoprotein 11 (*Con*Pgp-11) in the IVM-resistant CoIVR08 isolate compared to the IVM-susceptible CoIVSus isolate, both in vivo and in vitro [25]. Treatments with the Pgp inhibitor verapamil in jirds have demonstrated restoration of IVM sensitivity in ML-resistant *Haemonchus contortus* and *C. oncophora* isolates [26–28]. Similarly, *Parascaris* spp., a severely pathogenic intestinal nematode of equines, demonstrates genetic variations on *Parascaris equorum* P-glycoprotein 11 (*Peq*Pgp-11) transcripts that are correlated with reduced ML sensitivity [29]. Transgenic expression of *Peq*Pgp-11 has been shown to modulate IVM sensitivity in *Cel*Pgp-11 knockout *C. elegans* strains [30]. The localization, expression levels and genetic changes on Pgp-11 in *C. elegans, H. contortus, C. oncophora* and *P. equorum* indicate Pgp-11 is likely playing a functional role in the modulation of ML susceptibility and the development of resistance in these nematodes.

The characterization of *Dim*Pgp-11 is of particular interest in order to better understand the development of ML resistance in *D. immitis*. Here, we report an extensive study on the expression of *Dim*Pgp-11 and its localization in *D. immitis* microfilaria (mf).

## Methods

### *Dirofilaria immitis* isolates

Eight USA laboratory-maintained *D. immitis* isolates, collected from canine blood samples, were used to complete the various experiments (Table 1). The *D. immitis* samples were obtained while host dogs were not under drug pressure. The ML-susceptible isolates Missouri, MP3 and Yazoo isolates and the ML-resistant Metairie isolate were provided by the Filariasis Research Reagent Resource Center (FR3) [31]. The ML-susceptible isolate Berkeley, the putative ML-susceptible isolates Georgia III and Big Head and the ML-resistant isolate JYD-34 were provided by TRS Laboratories [31–33]. The sample providers, the FR3 and TRS laboratories, have the appropriate ethical permissions to house the nematodes at all stages of the life-cycle, including the mosquito colonies and canine definitive hosts. McGill University, where the experiments were conducted, does not house the all stages of the *D. immitis* life-cycle, and maintains the required biohazard importation permits from the Canadian Food Inspection Agency (CFIA Permit: A-2022-00829-1) to receive and work with the canine blood samples containing *D. immitis* mf.

**Table 1:** Laboratory-maintained *Dirofilaria immitis* isolates used or cited in the various experiments (MiSeq analysis, droplet digital PCR, immunofluorescence assay)

*D. immitis* isolate	Origin in the USA	Laboratory maintenance (duration)	Known/putative ML resistance phenotypic status
Missouri^a,b,c^	Puxico, MO	2005	Susceptible
MP3^a^	Athens, GA	2011	Susceptible
Yazoo^a^	Yazoo, MS	2017	Susceptible
Berkeley^b^	Berkeley County, SC	2014	Susceptible
Georgia II^a^	Vidalia, GA	2013	Susceptible
GCFL^a^	Fort Myers, FL	2014	Susceptible
ZoeAL^a^	Westover, AL	2015	Susceptible
Georgia III ^b^	Oconee County, GA	2017	Putative susceptible
Big Head^b^	Livonia, LA	2015	Putative susceptible
JYD-34^ab^	Keytesville MO	2010	Resistant
Metairie^a^	Metairie, LA	2017	Resistant
WildCat^a^	West Liberty, KY	2012	Resistant
ZoeAMAL^a^	Westover, AL	2014	Resistant

*ML* Macrocyclic lactone

^a^MiSeq analysis

^b^Droplet digital PCR (ddPCR) constitutive expression analysis

^c^Immunofluorescence assay

### Isolation of *D. immitis* mf

The canine venous blood samples were shipped overnight to McGill University for immediate processing. The mf were extracted from the blood by filtration as previously described [34]. The venous blood was diluted 1:1 with NaHCO_3_ solution, and the solution was filtered through polycarbonate membrane filters (3.0 µm; 25 mm; Sterlitech® Corporation; Auborn, WA, USA) to isolate mf. A modified Knott’s test was performed using 1 ml of blood.

### Measurement of *Dim*Pgp-11 genetic polymorphism

#### *Dim*Pgp-11 SNP marker

The *Dim*Pgp-11 SNP marker, found on *Dim*Pgp-11, scaffold nDi.2.2.scaf00004 at position 79,766, is one of 10 SNP markers validated in USA clinical samples [35]. We analyzed the ML-susceptible Missouri, MP3 and Yazoo isolates and the ML-resistant JYD-34 and Metairie isolates at this position, and compared the alternate allele frequency with results previously described in the USA laboratory-maintained isolates Berkeley, Georgia II, GCFL and ZoeAL (all ML-susceptible isolates) and WildCat and ZoeAMAL (both ML-resistant isolates) to confirm the continued association of the *Dim*Pgp-11 SNP with phenotypic ML resistance (Table 2) [33, 36].

**Table 2:** The alternate allele frequency of the *D. immitis* P-glycoprotein 11 single nucleotide polymorphic marker, scaffold nDi.2.2.scaf00004 at position 79766, in the ML-susceptible isolates Missouri, MP3, Yazoo, Berkeley, Georgia II, GCFL and ZoeAL and in the ML-resistant isolates JYD-34, Metairie, WildCat and ZoeAMAL, compared to the *D. immitis* reference genome nDi.2.2

*D. immitis* isolate	ML-susceptibility phenotype	Alternate allele frequency (%)
Missouri	Susceptible	12
MP3	Susceptible	0
Yazoo	Susceptible	0
Berkeley^a^	Susceptible	15
Georgia 2^a^	Susceptible	6
GCFL^b^	Susceptible	4
ZoeAL^b^	Susceptible	3
JYD-34	Resistant	40
Metairie	Resistant	36
WildCat^a^	Resistant	56
ZoeAMAL^b^	Resistant	17

^a^MiSeq analysis performed in 2021 [32]

^b^MiSeq analysis performed in 2017 [35]

#### Sample processing and DNA extraction

The genomic DNA (gDNA) from the total pooled mf of each isolate sample, ranging from 25,000–150,000 mf, was extracted using the QIAamp® DNA Micro kit (Qiagen Inc., Hilden, Germany). DNA concentrations were determined with the Quant-iT™ PicoGreen DNA Assay Kit (Invitrogen®, Life Technologies Inc., Thermo Fisher Scientific, Waltham, MA, USA). The samples were stored at − 80 ºC prior to being sent to Génome Québec for quality control and MiSeq Illumina sequencing (MiSeq System; Illumina Inc., San Diego, CA, USA).

#### MiSeq Illumina sequencing

The gDNA samples were sent to Génome Québec, and the regions encompassing the *Dim*Pgp-11 SNP were sequenced on an Illumina MiSeq platform, at a coverage of 2000×. Target enrichment was performed on the Access Array system (Fluidigm Corp. [now Standard BioTools Inc.], South San Francisco, CA, USA) using array-based PCR amplification of the genomic target region. The samples underwent parallel amplification using custom primers with added CS1 and CS2 tails, as described in [35]. The samples were barcoded during target enrichment, which allowed for multiplexed sequencing, and adapter sequences were added during the PCR amplification.

#### MiSeq Illumina sequencing data analysis

The reading tool Trimmomatic was used to trim the MiSeq Illumina sequencing data for minimal trailing quality (Phred score: 30) and to filter for minimum read length by removing the Illumina sequencing adapters from read and adapter clippings [37]. The resulting read pairs were aligned to the *D. immitis* reference genome nDi.2.2 (http://www.nematodes.org/genomes/dirofilaria_immitis) using BWA-mem (http://bio-bwa.sourceforge.net/), resulting in binary alignment map (BAM) files [38]. The alignments were processed with Picard (https://broadinstitute.github.io/picard) for the realignment of indels, mate fixing and marking of duplicate reads. BVATools (https://bitbucket.org/mugqic/bvatools/src) was used to extract base frequencies for the *Dim*Pgp-11 SNP, and the read frequencies were assimilated to the allele frequencies. The alternate allele variance of all samples were compared to the *D. immitis* reference genome nDi.2.2. The BAM files for the *D. immitis* Missouri, MP3, Yazoo, JYD-34 and Metairie isolates are openly available on the NCBI Sequence Read Archive under BioProject PRJNA847600.

### Quantification of *Dim*Pgp-11 constitutive expression

#### Extraction of *D. immitis* mf RNA

The RNA extraction, primer optimization and constitutive expression quantification protocols were optimized with the ML-susceptible Missouri isolate. The optimized protocol was further validated using the Missouri isolate and the ML-susceptible Berkeley isolate, the putative ML-susceptible Georgia III and Big Head isolates and the ML-resistant JYD-34 isolate [31–33].

The RNA extraction protocol is a modified TRIZol protocol used to accommodate the fracture-resistant cuticle of the *D. immitis* mf. Frozen samples of 100,000 mf were thawed and centrifuged for 5 min at high speed. The phosphate-buffered saline (PBS) supernatant was removed, and the mf were washed twice with 0.1× TE buffer. All subsequent work was performed in a fume hood. Samples were resuspended in a 1:4 ratio of 0.1× TE buffer and TRIzol™ LS reagent (Thermo Fisher Scientific), following which the samples were flash frozen in liquid N_2_ and broken down with a plastic pestle three times thrice. Acid-washed glass beads (200 µl; diameter: 425–600 µm; Sigma-Aldrich, St. Louis, MO, USA) were added to each sample, and then the samples were vortexed for 1 min and flash frozen in liquid N_2_ 5 times. A 100-µl aliquot of chloroform was then added and the samples vortexed for 15 s. Samples were incubated at room temperature for 5 min and then centrifuged at 12,000*g* for 5 min at 4 °C. The supernatant was recovered in a Phasemaker™ tube (Invitrogen®, Life Technologies Inc., Thermo Fisher Scientific) and centrifuged again at 12,000*g* at 4 °C for 15 min. The aqueous phase was transferred to a sterile Eppendorf tube, 250 µl of ice-cold isopropanol was added to the tube and the samples were then centrifuged at 12,200 *g* for 30 min at 4 °C. The samples were then stored at − 20 °C overnight for RNA precipitation.

After the overnight storage at − 20 °C, the samples were centrifuged at 12,200*g* for 30 min at 4 °C, and the supernatant was removed. The RNA pellet was washed with 1 ml of 75% ethanol and centrifuged twice at 12,200*g* for 10 min at 4° C. The ethanol supernatant was removed, and the samples dried in a fume hood for several hours until all remaining ethanol had evaporated. The RNA pellet was then resuspended in 0.1× TE buffer and incubated at 55° C to resolubilize. The RNA samples were treated with the Invitrogen DNA-free kit (Invitrogen®, Life Technologies Inc., Thermo Fisher Scientific) following the manufacturer’s instructions. The RNA concentration was assessed on a NanoDrop™ One spectrophotometer (Thermo Fisher Scientific), and the RNA quality was determined on native agarose gel. Complementary DNA (cDNA) was obtained by reverse transcription using the SuperScript™ VILO™ mix (Thermo Fisher Scientific). Prepared cDNA was serially diluted for droplet digital (ddPCR) analysis and frozen at − 80 °C.

#### ddPCR primer design

The PCR primers for *Dim*Pgp-11 and the three reference genes were prepared using NCBI Primer-BLAST (Additional file 1: Table S1). The nucleotide sequences and primers were blasted against the entire *D. immitis* genome. The primer design parameters included the following: PCR size between 70 and 150 bp, forward and reverse primer melting temperature (T_M_) difference ≤ 3° C, “GC” content ≥ 40%, T_M_ between 50—65° C, and no bp ≥ 3. Three sets of primers were prepared and tested for each gene. Primer validation was performed by quantitative PCR (qPCR) melt curve and ddPCR thermogradient.

#### Quantification of* Dim*Pgp-11 constitutive expression by ddPCR

*Dim*Pgp-11 constitutive expression quantification was determined using ddPCR. The samples were prepared in three biological replicates and plated in two technical replicates per isolate. The ddPCR master mixes, prepared for *Dim*Pgp-11 and the three reference genes, *Dim*GAPDH, *Dim*Actin, *Dim*pmp-3, comprise the QX200™ ddPCR™ EvaGreen Supermix (Bio-Rad Laboratories, Hercules, CA, USA), the forward and reverse primers, cDNA sample and nuclease-free water. The PCR master mix of each sample was mixed with droplet generation oil (Bio-Rad Laboratories) and partitioned into 20,000 nanoliter-sized droplets by a QX200™ Droplet Generator (Bio-Rad Laboratories). The new sample emulsions were plated on a 96—well plate using a multichannel pipette and heat sealed with a foil heat seal to undergo standard PCR amplification (Additional file 2: Table S2). Following the PCR amplification, each sample was read with the QX200™ Droplet Reader (Bio-Rad Laboratories) to determine the target concentration using Poisson’s statistics.

#### Statistical analysis of* Dim*Pgp-11 constitutive expression

The ddPCR results of *Dim*Pgp-11 constitutive expression were analyzed by normalizing copy numbers against the geometric mean of the copy numbers of the three reference genes: *Dim*GAPDH, *Dim*Actin and *Dim*pmp-3. All experiments were performed in three biological replicates. All data are reported as mean fold change, with error bars corresponding to the standard deviation of three biological replicates. The replicates for the five isolates were prepared at a concentration of 100,000 mf. The known ML-susceptible Missouri isolate was used as the one-fold expression standard. The significance of the fold changes in constitutive expression were compared to the Missouri isolate by the parametric unpaired t-test with Welch correction, two-tailed* P*-value and 95% confidence interval using Prism 9.3.0c (GraphPad Software, Inc., San Diego, CA, USA).

### *Dim*Pgp-11 immunofluorescence assay

#### Antibody design and validation

**Fig. 1:**
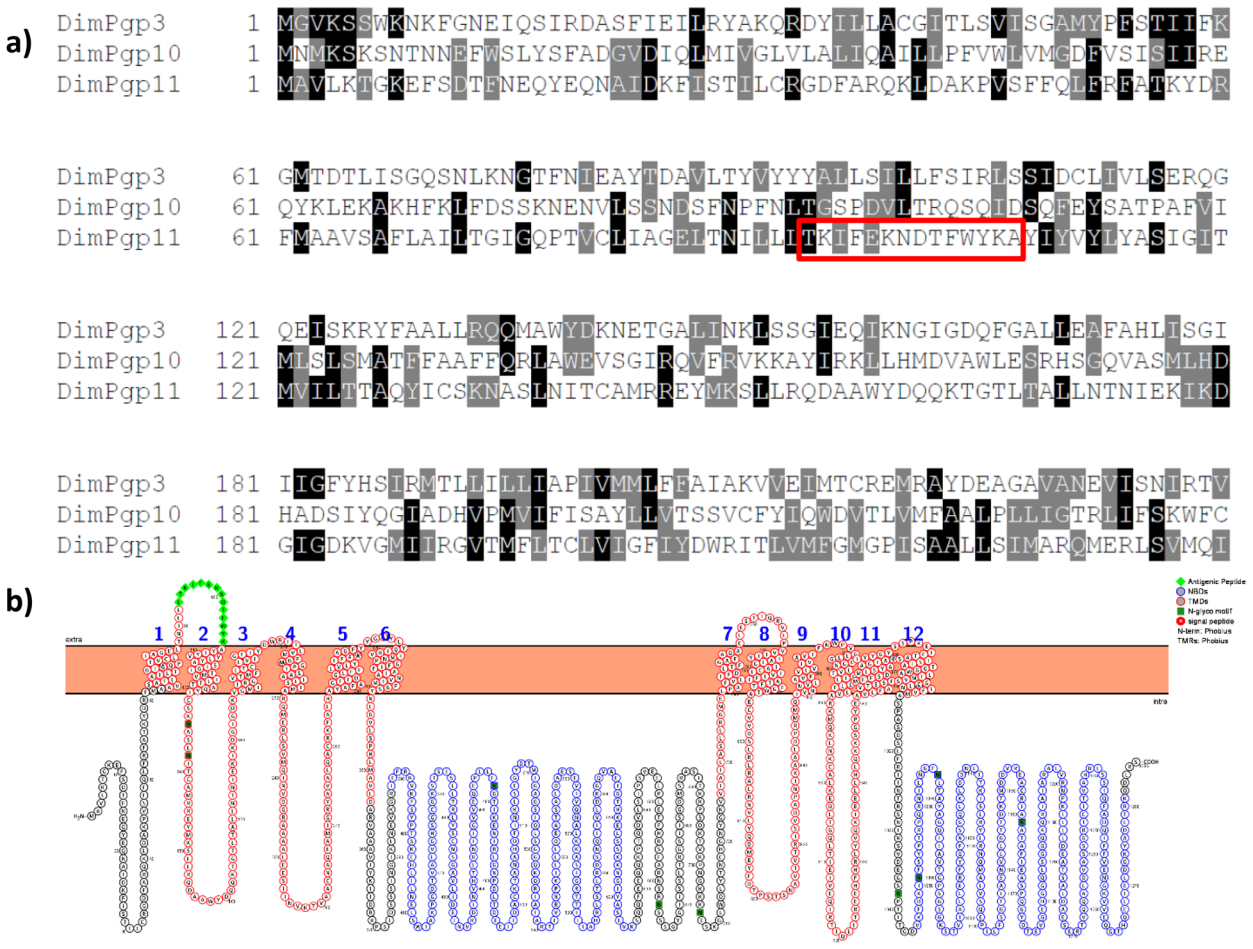
**a** Multiple sequence alignment of *Dim*Pgp-3, *Dim*Pgp-10 and *Dim*Pgp-11 amino acid sequences. The protein regions chosen as the antigenic determinant are highlighted in red. Visualization was prepared using the ExPASy BoxShade (https://embnet.vital-it.ch/software/BOX_form.html). **b** Two-dimensional visualization of *Dim*Pgp-11. The sequence of the transmembrane domains (TMDs), TMD1 (residues 64–354) and TMD2 (residues 726–1012), are framed in red. The nucleotide binding domains (NBDs), NBD1 (residues 389–625) and NBD2 (residues 1045–1218), are framed in blue. Putative N-glycosylation motifs (Asn 100, 134, 138, 478, 663, 706, 1051, 1067, 1161) are highlighted in dark green. The peptide region chosen as the antigenic determinant, the first extracellular loop of the first transmembrane domain, is highlighted in light-green diamonds. Visualization was prepared using the Protter interactive protein visualization tool. * Dim*Pgp 3, 10, 11, *Dirofilaria immitis* P-glycoprotein 3, 10, 11, respectively;

The amino acid sequences of the three *D. immitis* Pgps *Dim*Pgp-3, *Dim*Pgp-10 and *Dim*Pgp-11 were collected from the WormBase ParaSite [39–41]. Multiple sequence alignment of the *D. immitis* Pgps was performed using ExPASy–BoxShade [42] (Fig. 1a). The topology of the full-length *Dim*Pgp-11 protein sequence across the membrane bilayer was visually represented using Protter [43]. The transmembrane helices and nine N-glycosylation motifs were represented as predicted by the program.

The peptide target of the *Dim*Pgp-11 antigen affinity antibody, LTKIFEKNDTFWYK, located in the first extracellular loop of the first transmembrane domain (TMD) between helices 1 and 2, was isolated (Fig. 1b). The chosen sequence was blasted against the entire *D. immitis* genome to confirm the sequence specificity to *Dim*Pgp-11. All information, including *Dim*Pgp-11 molecular weight, which was approximately 140 kDa, was reported to the GenScript Biotech Corporation (Piscataway, NJ, USA) which first analyzed the suitability and specificity of the antigen and then prepared an antibody that indicated high specificity to the target peptide. The antigen affinity antibody was further validated via western blot and competitive binding assay in *D. immitis* membrane protein lysate and a HEK cell membrane protein lysate control (Additional file 3: Fig S1).

#### Immunofluorescence assay

*Dirofilaria immitis* mf were fixed and permeabilized following an adaption of the WormBook Immunohistochemistry tube fixation protocol for *C. elegans*, as described in [44]. The mf were centrifuged for 5 min at 1000 *g*. Freeze-cracking was performed in a fixing solution (3.7% wt/vol paraformaldehyde in PBS) by placing the samples in liquid N_2_ for 2 min and thawing 3 times in a 37 °C water bath. The samples were incubated in the paraformaldehyde fixative on a shaker for 4 h at 4° C, followed by centrifugation and three washes with PBST (0.1% Triton X—100 in PBS). To permeabilize the mf cuticle, the samples were resuspended in fresh β—mercaptoethanol and incubated overnight at 37° C. The samples were then washed extensively in PBST. Worms were incubated for 3 h in collagenase solution (1000 U/ml collagenase type XI; Sigma-Aldrich), 1 mM CaCl_2_, 0.1% Triton X—100, 100 mM Tris, pH 7.4) at 37 °C. The mf were washed with PBST and then incubated overnight with antibody diluent solution (AbD) (0.1% bovine serum albumin, 0.1% sodium azide in PBST).

Incubation of the samples with the *Dim*Pgp-11 primary affinity-purified antibody in AbD (1:100; GenScript Biotech Corporation) was performed for 3 days at 4° C. The unbound primary antibody was removed by several washes in AbD and the samples incubated overnight at 4° C. The incubation with secondary AlexaFluro 488 antibody diluted in AbD (1:1000; Invitrogen®, Life Technologies Inc., Thermo Fisher Scientific) was performed for 12 h at 4° C. The unbound secondary antibody was removed by several washes in AbD and the samples incubated overnight at 4 °C. Rhodamine-phalloidin (200 ng/ml; Cytoskeleton Inc., Denver, CO, USA) and DAPI (50 ng/ml; Sigma-Aldrich) were used to counterstain the muscle tissue and cell nuclei, respectively, by overnight incubation. The samples were washed in AbD and incubated overnight. The fully stained mf samples were fixed in Mounting Media (Sigma-Aldrich) on slides. The slides were observed on a Nikon A1R MP confocal microscope (Nikon Corp., Tokyo, Japan; Additional file 4: Fig S2; Additional file 5: Fig S3). The control observation of mf with omission of primary antibody was also tested (Additional file 6: Fig S4).

## Results

### *Dim*Pgp-11 genetic polymorphism in IVM-resistant *D. immitis*

The SNP marker on *DimPgp-11* was analyzed in the ML-susceptible *D. immitis* Missouri, MP3 and Yazoo isolates and the *D. immitis* ML-resistant JYD-34 and Metairie isolates. The alternate allele frequency ranged from 0% to 12% in the three ML-susceptible isolates, and from 36% to 40% in the two resistant isolates (Table 2). Among the previously described USA laboratory-maintained isolates, the alternate allele frequency ranged from 0% to 15% in the ML-susceptible isolates Berkeley, Georgia II, GCFL and ZoeAL, and from 17% to 56% in the ML-resistant isolates WildCat and ZoeAMAL (Table 2) [33, 36]. The MiSeq results for the ML-susceptible Missouri, MP3 and Yazoo isolates and for the ML-resistant JYD-34 and Metairie isolates fell within the range of those for the previously described USA laboratory-maintained isolates.

### PCR primer design and validation of ddPCR

The primers for *Dim*Pgp-11 and the three reference genes were tested and validated using the known ML-susceptible Missouri isolate [31]. Three sets of primers were designed for each gene., and each set was first tested using qPCR. cDNA dilutions were prepared at 1:1, 1:5, 1:25 and 1:125. Those with a single melting curve, indicating amplification of a single discrete product, were subsequently validated with ddPCR. The cDNA dilutions utilized in the ddPCR analysis were chosen based on the qPCR cycle. The ddPCR thermogradient cycle ranged from 51 °C to 63 °C and was utilized to determine the ideal annealing temperature. The *Dim*Pgp-11 primers were designed to not include the SNP found in the second transmembrane domain. The ideal annealing temperature for the *Dim*Pgp-11 primer set was 55.7 °C (Additional file 2: Table S2; Additional file 7: Fig S5).

The three housekeeping genes utilized in ddPCR analysis were *Dim*Actin, *Dim*GAPDH and *Dim*pmp-3. *Dilofilaria immitis* currently does not have a set of known high-quality reference genes. Six genes were analyzed to choose the optimal reference genes: Actin, GAPDH, pmp-3, Histone H3, CDC42 and Y45F10D.4. These genes were chosen because they have been previously validated using multi-software as high-quality reference genes for *C. elegans* and *Brugia malayi* via geNorm, NormFinder, BestKeeper and Comparative ΔCt [45–47]. Three sets of primers were prepared for each of the six genes. All six genes underwent qPCR melt curve analysis. Of the six genes tested, four had clean melt curves in the *D. immitis* samples: *Dim*Actin, *Dim*GAPDH, *Dim*pmp-3 and *Dim*Histone H3. The four potential reference genes then underwent ddPCR thermogradient analysis, and demonstrated clean droplet separation, indicating there was no non-specific amplification. The *Dim*Actin, *Dim*GAPDH and *Dim*pmp-3 housekeeping genes were selected at an annealing temperature of 58.4 °C. *Dim*Histone H3 had an ideal annealing temperature at 51° C and was not used in subsequent analysis (Additional file 4: Fig S2).

### Quantification of *Dim*Pgp-11 constitutive expression

**Fig. 2:**
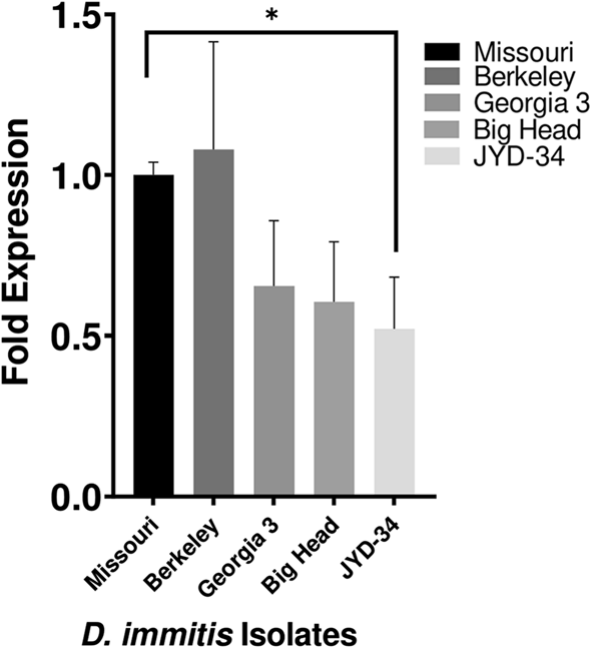
Constitutive expression of *D. immitis* Pgp-11 in the mf life-stage of the ML-susceptible Missouri and Berkeley isolates, the putative susceptible Georgia III and Big Head isolates and the ML-resistant JYD-34 isolates was determined using droplet digital PCR. The changes in constitutive expression were calculated relative to the ML-susceptible Missouri isolate, set as the onefold control. The copy numbers of* Dim*Pgp-11 were normalized to the copy numbers of the three reference genes:* Dim*GAPDH,* Dim*Actin and* Dim*pmp-3. All data are reported as mean fold change, with error bars corresponding to the standard deviation of three individual replicates. The three biological replicates of each of the five USA laboratory-maintained isolates were prepared at a concentration of 100,000 mf. The significance of the constitutive expression fold changes relative to the Missouri isolate was analyzed by the parametric unpaired t-test with Welch correction, two-tailed* P*-value and 95% confidence interval using Prism 9.3.0c (GraphPad Software, Inc.). Asterisk indicates a significant difference (*P* = 0.03) in the constitutive expression of *Dim*Pgp-11 between the JYD-34 and Missouri isolates

Constitutive expression of *Dim*Pgp-11 was measured in the mf from five *D. immitis* isolates: the ML-susceptible Missouri and Berkeley isolates, the putative susceptible Georgia III and Big Head isolates and the ML-resistant isolate JYD-34 [31–33]. The known ML-susceptible Missouri isolate was set as the onefold standard as it is a well-characterized ML-susceptible isolate [31]. The fold expression reported is the mean fold change of three biological replicates (Fig. 2). When compared to the Missouri isolate reference, the ML-susceptible Berkeley isolate and the two putative-susceptible isolates did not show statistically significant differences in the constitutive expression of *Dim*Pgp-11 (Fig. 2). Interestingly, the ML-resistant JYD-34 isolate demonstrated a statistically significant decrease in *Dim*Pgp-11 constitutive expression when compared to the ML-susceptible Missouri isolate (*P* = 0.03; Fig. 2).

### *Dim*Pgp-11 immunofluorescence assay

**Fig. 3:**
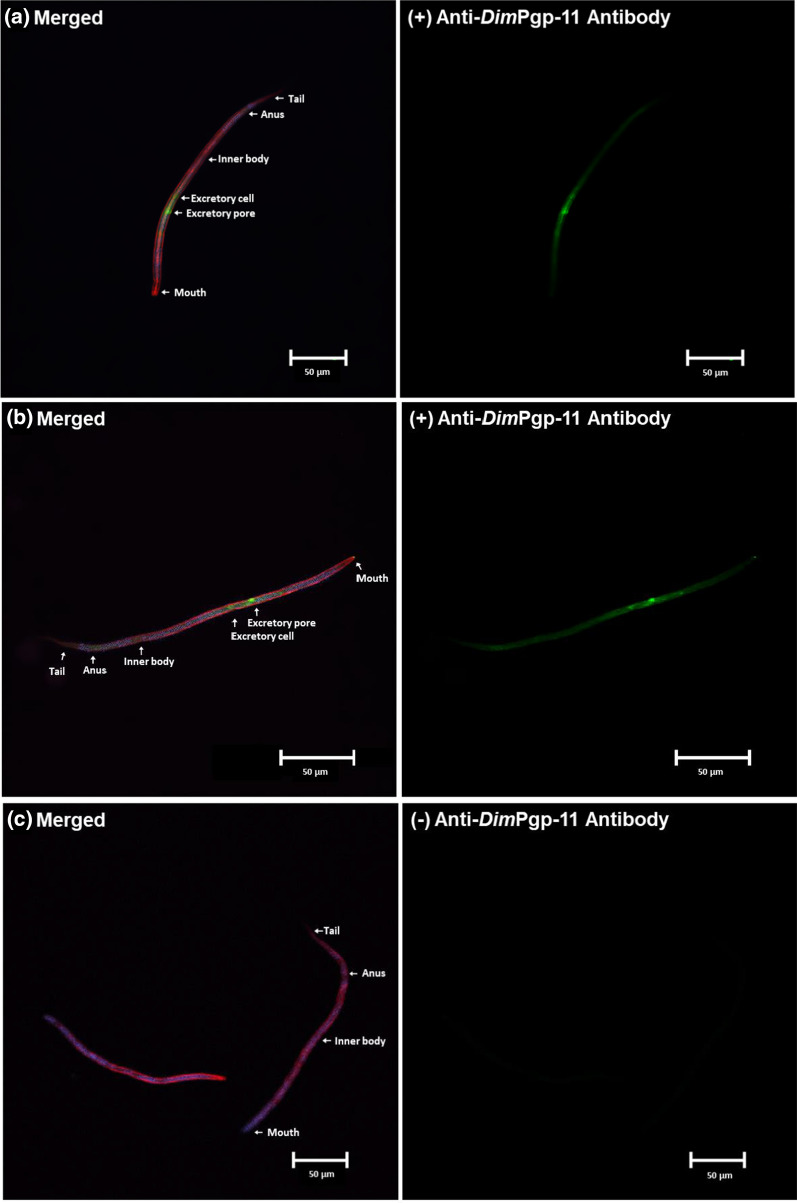
Immunofluorescence assay of DimPgp-11 in Dirofilaria immitis mf by confocal laser microscopy. DimPgp-11 specific signals detected in proximity to the ESP, excretory cell and faintly within the body wall with primary DimPgp-11 antigen affinity antibody and the secondary AlexaFluro488 antibody (green). Counterstaining of Actin with Rhodamine-Phalloidin (red), and cell nuclei with Dapi (blue) distinguished major anatomical features such as the mouth, excretory pore, excretory cell, inner body, anus, and tail in merged immunofluorescence images. **a** DimPgp-11 localization in Worm 1 and **b** DimPgp-11 localization in Worm 2. **c** Non-primary antibody control. The non-primary antibody control was incubated in antibody diluent rather than the primary DimPpg-11 antigen affinity antibody and then incubated with secondary AlexaFluro488 antibody. The absence of immunostaining confirms the specificity of the DimPgp-11 signal observed in Worm 1(**a**) and 2(**b**)

The validated polyclonal antibody was used in an immunofluorescence assay (IFA) to localize *Dim*Pgp-11 in the mf life-stage of the ML-susceptible Missouri isolate. The mf were counterstained with rhodamine-phalloidin and DAPI, which distinguished major anatomical figures, such as the mouth, excretory pore, excretory cell, inner body, anus and tail (Fig. 3; Additional file 4: Fig S2, Additional file 5: Fig S3). *Dim*Pgp-11 was strongly expressed in the pore and duct cells which surround the excretory-secretory pore (ESP), the excretory cell and, more faintly, along the mf body wall*.*

## Discussion

The SNP changes on *Dim*Pgp-11 between the ML-susceptible and ML-resistant *D. immitis* isolates were one of the first genetic markers of ML resistance [15]. *Dim*Pgp-11 was among the top 10 SNP markers validated in US clinical samples and was similarly one of the 42 nodes used to assess the genetic profiles of 10 *D. immitis* isolates with known ML-susceptible or ML-resistant phenotypes to dirofilariosis preventives [35, 36]. In the current study, we have confirmed differences in the frequency of the alternative nucleotide on scaffold nDi.2.2.scaf00004 at position 79766, *Dim*Pgp-11 SNP, between USA laboratory-maintained ML-susceptible and ML-resistant isolates. The ML-resistant isolates showed higher alternate allele frequencies in comparison to the reference genome and the ML-susceptible isolates [33, 36].

Our results demonstrate that there are genotypic differences in the *Dim*Pgp-11 gene between ML-susceptible and ML-resistant USA laboratory maintained *D. immitis* isolates. However, the gene sequence and the presence of the *Dim*Pgp-11 SNP marker do not predict the level of expression that can possibly also alter protein activity. We assessed the constitutive expression of *Dim*Pgp-11 in ML-susceptible and ML-resistant USA laboratory-maintained *D. immitis* isolates via ddPCR analysis. The lower level of *Dim*Pgp-11 constitutive expression in the ML-resistant JYD-34 isolate, when compared to the ML-susceptible Missouri isolate, suggests a possible change in the activity of *Dim*Pgp-11. It is an indication that Pgps are an important factor in ML resistance, as previously reported in other nematodes of veterinary importance nematodes, even if changes in Pgp expression associated with ML resistance are generally an overexpression of the Pgps [23–25]. Gene transcription, however, is not always a one-to-one indication of expression levels or protein activity. The documented genetic changes on *Dim*Pgp-11, such as the *Dim*Pgp-11 SNP, may impact the configuration of the ATP binding sites, and drug efflux activity, possibly changing transporter activity. Conversely, analysis of additional ML-resistant isolates as they become available to academic labs may provide increased clarity on whether this reduction in constitutive expression is associated with an ML-resistant genotype or whether it is specific to the JYD-34 isolate, but not specifically associated with resistance.

The *Dim*Pgp-11 protein was localized in the ML-susceptible Missouri isolate within the ESP/duct cells and excretory cell and, more faintly, within the mf body wall. The strategic location of *Dim*Pgp-11 making up the excretory-secretory system suggests the Pgp may play an active role in regulating ML in the mf ESP.

Since ML resistance in *D. immitis* may depend on several factors, we do not yet fully understand the genetics of ML resistance in *D. immitis*, and a number of genes may make variable contributions to a resistance phenotype. Within a single host producing mf, there will be a number of individual heartworms, each with its own genotype. Thus, *D. immitis* populations are genetically diverse, unlike populations of drug-resistant clonal organisms, such as bacteria. Phenotypic susceptibility, using a single ML preventive at a single dose rate, may not be a clear indication of the ML susceptibility status of each individual worm in a population. Evidence for developing resistance may only become apparent, in vivo, if dose–response studies are performed. There may be *D. immitis* populations which appear susceptible at a given ML dose rate but may show a shift in susceptibility if a dose–response curve is investigated. Recent results from enzymatic, MiSeq, ddPCR duplex and microsatellite studies have demonstrated that the current definitions used to define “ML-susceptible” and “ML-resistant” isolates lack nuance [48, 49]. Apparent phenotypic ML susceptibility, at a given preventive dose regime, may not correlate perfectly with genotypic ML susceptibility due to the heterologous nature of the population. *Dim*Pgp-11 is possibly one of several genes linked with the development of ML resistance in *D. immitis.* By considering the heterologous *D. immitis* populations and the nature of multigenic drug resistance, *Dim*Pgp-11 may be the first of multiple genes used to assess the genotypic variability and distinguish ML-susceptible, genetically mixed and ML-resistant isolates.

## Conclusion

The first genetic changes found to differentiate ML-susceptible and ML-resistant isolates were SNPs on *Dim*Pgp-11, and this gene remains of interest in understanding ML resistance in *D. immitis*. Constitutive expression of *Dim*Pgp-11 in the ML-resistant JYD-34 isolate was significantly lower than that in a well-characterized ML-susceptible isolate. The transcription level does not always provide a one-to-one indication of protein activity and drug efflux. However, the site of expression of *Dim*Pgp-11 and the differences in constitutive expression between resistant and susceptible isolates suggest that *Dim*Pgp-11 may help regulate the ESP response to MLs, including the secretion of immunomodulatory molecules. Further analysis is required to review the multiple genetic changes found in the *Dim*Pgp-11 genetic sequence and how these changes may impact protein folding, the configuration of the ATP binding sites and drug efflux activity.

## Supplementary Information

**Additional file 1**: **Table S1.** Droplet digital PCR forward and reverse primer sequences for *Dirofilaria immitis* P-glycoprotein 11, and reference genes Actin, GAPDH and pmp-3.

**Additional file 2: Table S2.** Droplet digital PCR amplification cycle for constitutive expression quantification of *Dim*Pgp-11.

**Additional file 3**: **Figure S1. a** Western blot analysis of *Dirofilaria immitis* P-glycoprotein 11 (*D*) from whole membrane protein lysate using anti-*Dim*Pgp-11 1° polyclonal antigen affinity antibody (1:1000 dilution) and an α-rabbit HRP 2° antibody (1:5000 dilution) with a HEK cell whole membrane protein lysate (*H*) negative control. Bands shown are those located at *Dim*Pgp-11 molecular weight of approximately 140 kDa and two bands at approximately 70 kDa, the molecular half weight of the full-length transporter and likely proteolytic fragments of the full-length *Dim*Pgp-11.** b** Competitive ligand binding assay analysis of anti-*Dim*Pgp-11 1° polyclonal antigen affinity antibody tested with 5× incubation with immunizing peptide (LTKIFEKNDTFWYK), which shows the disappearance of the approximately 140-kDa full-length *Dim*Pgp-11 band and the half-size bands (likely proteolytic fragments).

**Additional file 4: Figure S2.** Immunofluorescence assay of *Dim*Pgp-11 in *Dirofilaria immitis* mf by confocal laser microscopy. *Dim*Pgp-11-specific signals were detected in proximity to the ESP, excretory cell and faintly within the body wall using the primary *Dim*Pgp-11 antigen affinity antibody and the secondary AlexaFluror 488 antibody (green). Actin was counterstained with rhodamine-phalloidin (red), and nuclei were counterstained with DAPI (blue). The counterstain controls distinguish major anatomical features, such as the mouth, excretory pore, excretory cell, inner body, anus and tail.

**Additional file 5: Figure S3.** Immunofluorescence assay of *Dim*Pgp-11 in *Dirofilaria immitis* mf by confocal laser microscopy. *Dim*Pgp-11-specific signals were detected in proximity to the ESP, excretory cell and faintly within the body wall using the primary *Dim*Pgp-11 antigen affinity antibody and the secondary AlexaFluror 488 antibody (green). Actin was counterstained with rhodamine-phalloidin (red), and nuclei were counterstained with DAPI (blue). The counterstain controls distinguish major anatomical features such as the mouth, excretory pore, excretory cell, inner body, anus and tail.

**Additional file 6: Figure S4.** Non-primary antibody control of immunofluorescence assay of *Dim*Pgp-11 in *Dirofilaria immitis* mf by confocal laser miroscopy. Samples were incubated in antibody diluent rather than primary *Dim*Ppg-11 antigen affinity antibody and the secondary AlexaFluro488 antibody. The absence of immunostaining confirms the specificity of the signal. Actin was counterstained with rhodamine-phalloidin (red), and nuclei were counterstained with DAPI (blue). The counterstain controls distinguish major anatomical features such as the mouth, excretory pore, excretory cell, inner body, anus and tail.

**Additional file 7: Figure S5.** Raw thermogradient (51–63 °C) constitutive expression data of:** a** *Dirofilaria immitis* Pgp-11,** b** *D. immtis* GAPDH,** c** *D. immitis* Actin, **d** *D. immitis* pmp-3,** e** *D. immitis* Histone H3, in the Missouri isolate, determined using droplet digital PCR

## Data Availability

The data presented in this study is included within the article. Bioinformatic files for the Missouri, MP3, Yazoo, JYD-34 and Metairie *Dirofilaria immitis* isolates, i.e. SAMN28946274, SAMN28946275, SAMN28946276, SAMN28946277 and SAMN28946278, are openly available on the NCBI Sequence Read Archive as BAM files under BioProject PRJNA847600.

## Abbreviations

AbD: Antibody diluent
BAM: Binary alignment
cDNA: Complementary DNA
*Cel*: *Caenorhabditis** elegans* P-glycoprotein 11
CFIA: Canadian Food Inspection Agency
*Con*Pgp-11: *Cooperia oncophora* P-glycoprotein 11
ddPCR: Droplet digital PCR
*Dim*Pgp-3: *Dirofilaria immitis* P-glycoprotein 3
*Dim*Pgp-10: *Dirofilaria immitis* P-glycoprotein 10
*Dim*Pgp-11: *Dirofilaria immitis* P-glycoprotein 11
ESP: Excretory-secretory pore
FR3: Filariasis Research Reagent Resource Center
gDNA: Genomic DNA
IFA: Immunofluorescence assay
IVM: Ivermectin
LOE: Loss of efficacy
mf: Microfilariae
ML: Macrocyclic lactones
NBD: Nucleotide binding domain
PBS: Phosphate-buffered saline
PBST: Phosphate-buffered saline with Triton—X 100
*Peq*Pgp-11: *Parascaris equorum* P-glycoprotein 11
Pgp: P-glycoprotein
qPCR: Quantitative PCR
SNP: Single nucleotide polymorphism
T_M_: Melting temperature
TMD: Transmembrane domain
