# Supplementary material for: Genetic polymorphism, constitutive expression and tissue localization of Dirofilaria immitis P-glycoprotein 11: a putative marker of macrocyclic lactone resistance
Source: Parasit Vectors. 2022 Dec 21;15:482. doi: 10.1186/s13071-022-05571-6 (PMC9773537; doi:10.1186/s13071-022-05571-6)
Supplement: Supplementary file 1 — Additional file 1: Table S1. Droplet digital PCR forward and reverse primer sequences for Dirofilaria immitis P-glycoprotein 11, and reference genes Actin, GAPDH and pmp-3. [file 13071_2022_5571_MOESM1_ESM.docx]

**Table S1.** Droplet digital PCR forward and reverse primer sequences for *Dirofilaria immitis* P-glycoprotein 11, and reference genes Actin, GAPDH, and pmp-3.

| **Gene** |  | **Primer Sequence** |
| --- | --- | --- |
| P-glycoprotein 11 | Forward | TACGTGAAGCTTGCAGAATA |
|  | Reverse | TTCTGTGTCATATCCCTGTG |
| Actin | Forward | GTTCACAACCACGGCAGAAC |
|  | Reverse | AAAGCTTCTGGGCATCGGAA |
| GAPDH | Forward | CGGCCACTCAGAAGACTGTT |
|  | Reverse | GTTGGCACACGAAAGCCAT |
| pmp-3 | Forward | TACTGTCCCATCCTCAGTAG |
|  | Reverse | TAAGTGACCTTGTTTGCCAT |
