# Supplementary material for: Genetic polymorphism, constitutive expression and tissue localization of Dirofilaria immitis P-glycoprotein 11: a putative marker of macrocyclic lactone resistance
Source: Parasit Vectors. 2022 Dec 21;15:482. doi: 10.1186/s13071-022-05571-6 (PMC9773537; doi:10.1186/s13071-022-05571-6)
Supplement: Supplementary file 2 — Additional file 2: Table S2. Droplet digital PCR amplification cycle for constitutive expression quantification of DimPgp-11. [file 13071_2022_5571_MOESM2_ESM.docx]

**Table S2.** Droplet digital PCR amplification cycle for transcript level quantification of *Dim*Pgp-11

| **Steps** | **1** | **2** | **3** | **4** | **5** | **6** | **7** | **8** |
| --- | --- | --- | --- | --- | --- | --- | --- | --- |
| Temp | 95°C | 95°C | 55.7 °C^a^ | 72°C | Go To Step 2 | 4°C | 90°C | 12°C |
| Time (min) | 5:00 | 0:30 | 1:00 | 0:30 | 49 X | 5:00 | 5:00 | ∞ |

^a^ 55.7 °C for *Dim*Pgp-11 samples; 58.4 °C for *Dim*Actin, *Dim*GAPDH, and *Dim*pmp-3
