# Supplementary material for: Genetic polymorphism, constitutive expression and tissue localization of Dirofilaria immitis P-glycoprotein 11: a putative marker of macrocyclic lactone resistance
Source: Parasit Vectors. 2022 Dec 21;15:482. doi: 10.1186/s13071-022-05571-6 (PMC9773537; doi:10.1186/s13071-022-05571-6)
Supplement: Supplementary file 3 — Additional file 3: Figure S1. a Western blot analysis of Dirofilaria immitis P-glycoprotein 11 (D) from whole membrane protein lysate using anti-DimPgp-11 1° polyclonal antigen affinity antibody (1:1000 dilution) and an α-rabbit HRP 2° antibody (1:5000 dilution) with a HEK cell whole membrane protein lysate (H) negative control. Bands shown are those located at DimPgp-11 molecular weight of approximately 140 kDa and two bands at approximately 70 kDa, the molecular half weight of the full-length transporter and likely proteolytic fragments of the full-length DimPgp-11. b Competitive ligand binding assay analysis of anti-DimPgp-11 1° polyclonal antigen affinity antibody tested with 5× incubation with immunizing peptide (LTKIFEKNDTFWYK), which shows the disappearance of the approximately 140-kDa full-length DimPgp-11 band and the half-size bands (likely proteolytic fragments). [file 13071_2022_5571_MOESM3_ESM.docx]

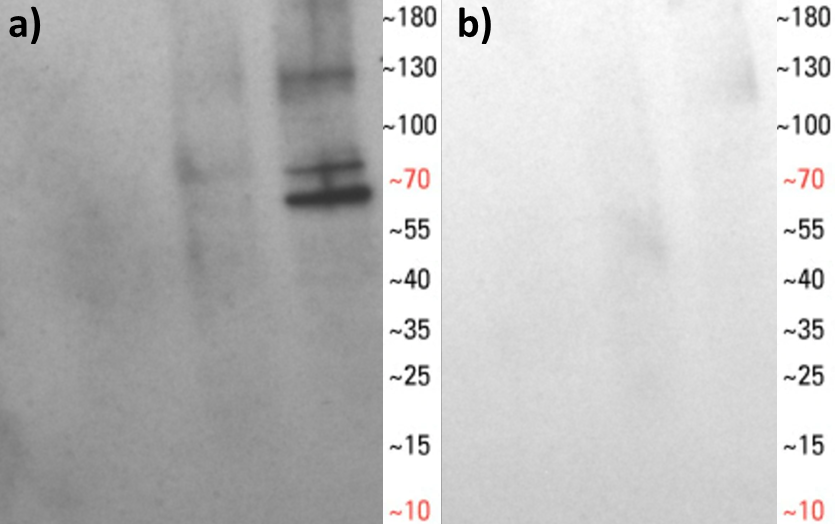


**D**

**D**

**H**

**H**

^D^ *Dirofilaria immitis* whole membrane protein lysate

^H^ HEK Cell whole membrane protein lysate

**Figure S1** a) Western Blot analysis of *Dirofilaria immitis* P-glycoprotein 11 (D) from whole membrane protein lysate using anti-*Dim*Pgp-11 1° polyclonal antigen affinity antibody (1 : 1000 dilution) and a α-rabbit HRP 2° antibody (1 : 5000 dilution) with a HEK cell whole membrane protein lysate (H) negative control. Bands located at *Dim*Pgp-11 molecular weight of ~140kDa and two bands at ~70kDA, the molecular half weight of the full-length transporter, and likely proteolytic fragments of the full-length *Dim*Pgp-11; b) Competitive ligand binding assay analysis of anti-*Dim*Pgp-11 1° polyclonal antigen affinity antibody tested with 5X incubation with immunizing peptide (LTKIFEKNDTFWYK), which shows the disappearance of the ~140kDA full length *Dim*Pgp-11 band and the half-size bands (likely proteolytic fragments).
