# Supplementary material for: Genetic polymorphism, constitutive expression and tissue localization of Dirofilaria immitis P-glycoprotein 11: a putative marker of macrocyclic lactone resistance
Source: Parasit Vectors. 2022 Dec 21;15:482. doi: 10.1186/s13071-022-05571-6 (PMC9773537; doi:10.1186/s13071-022-05571-6)
Supplement: Supplementary file 5 — Additional file 5: Figure S3. Immunofluorescence assay of DimPgp-11 in Dirofilaria immitis mf by confocal laser microscopy. DimPgp-11-specific signals were detected in proximity to the ESP, excretory cell and faintly within the body wall using the primary DimPgp-11 antigen affinity antibody and the secondary AlexaFluror 488 antibody (green). Actin was counterstained with rhodamine-phalloidin (red), and nuclei were counterstained with DAPI (blue). The counterstain controls distinguish major anatomical features such as the mouth, excretory pore, excretory cell, inner body, anus and tail. [file 13071_2022_5571_MOESM5_ESM.docx]

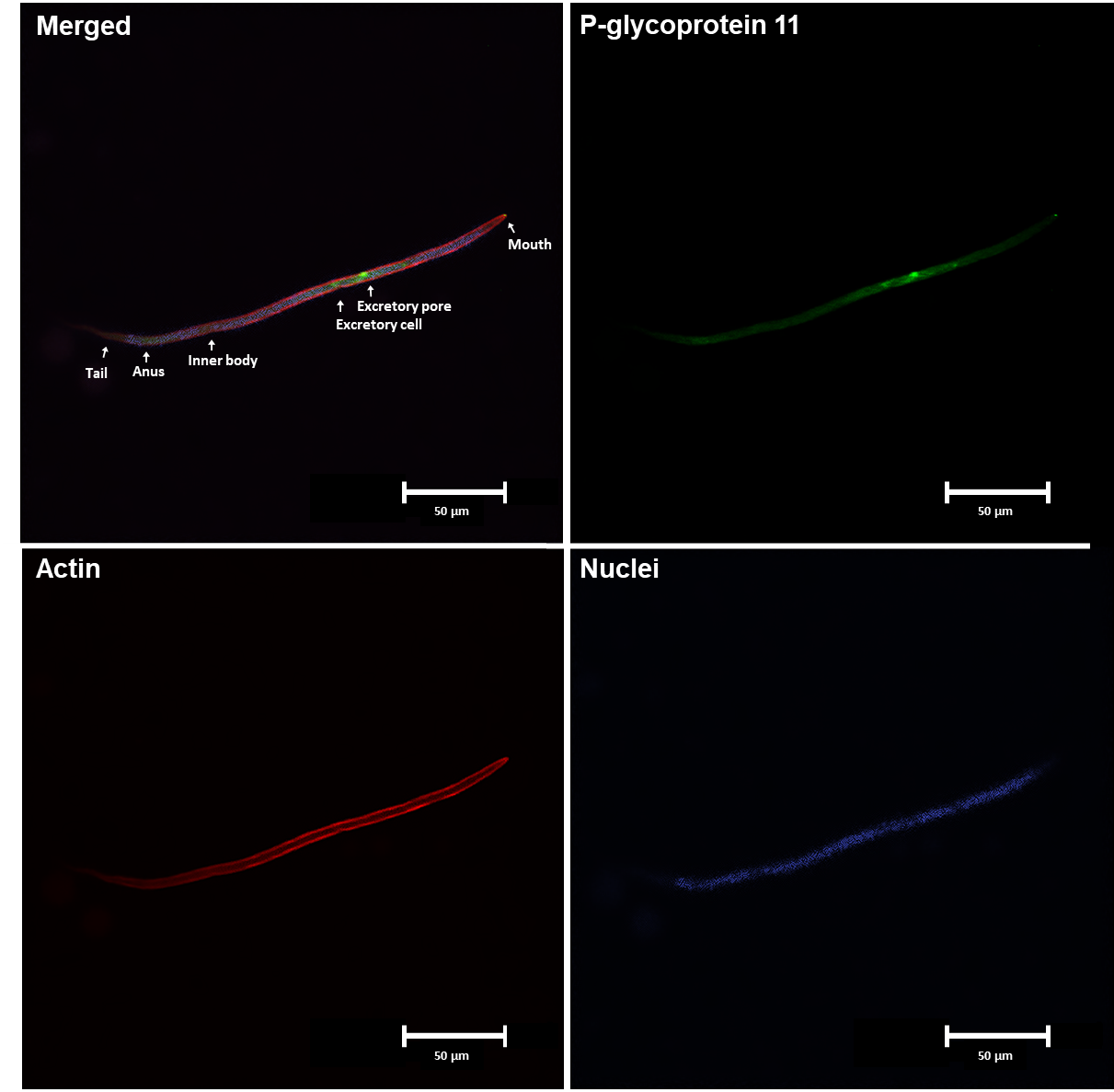


**Figure S4.** Immunofluorescence assay of *Dim*Pgp-11 in *Dirofilaria immitis* mf by confocal laser microscopy. *Dim*Pgp-11 specific signals was detected in proximity to the ESP, excretory cell and faintly within the body wall using the primary *Dim*Pgp-11 antigen affinity antibody and the secondary AlexaFluror 488 antibody (green). Actin was counterstained with Rhodamine-Phalloidin (red), and nuclei were counterstained with Dapi (blue). The counterstain controls distinguish major anatomical features such as the mouth, excretory pore, excretory cell, inner body, anus, and tail.
