# Supplementary material for: Genetic polymorphism, constitutive expression and tissue localization of Dirofilaria immitis P-glycoprotein 11: a putative marker of macrocyclic lactone resistance
Source: Parasit Vectors. 2022 Dec 21;15:482. doi: 10.1186/s13071-022-05571-6 (PMC9773537; doi:10.1186/s13071-022-05571-6)
Supplement: Supplementary file 6 — Additional file 6: Figure S4. Non-primary antibody control of immunofluorescence assay of DimPgp-11 in Dirofilaria immitis mf by confocal laser miroscopy. Samples were incubated in antibody diluent rather than primary DimPpg-11 antigen affinity antibody and the secondary AlexaFluro488 antibody. The absence of immunostaining confirms the specificity of the signal. Actin was counterstained with rhodamine-phalloidin (red), and nuclei were counterstained with DAPI (blue). The counterstain controls distinguish major anatomical features such as the mouth, excretory pore, excretory cell, inner body, anus and tail. [file 13071_2022_5571_MOESM6_ESM.docx]

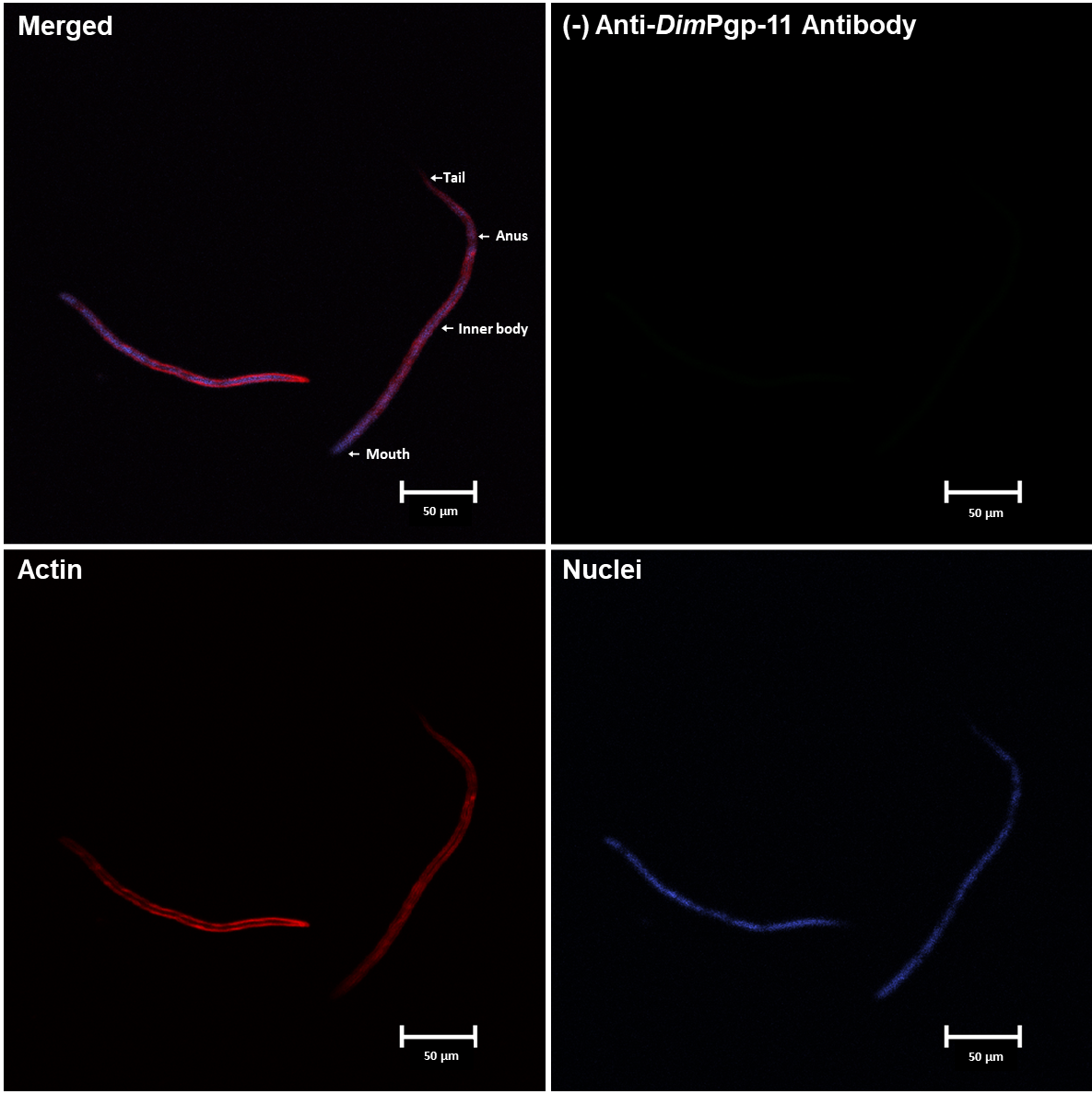


**Figure S5.** Non-primary antibody control of immunofluorescence assay of *Dim*Pgp-11 in *Dirofilaria immitis* mf by confocal laser miroscopy. Samples were incubated in antibody diluent rather than primary *Dim*Ppg-11 antigen affinity antibody and the secondary AlexaFluro488 antibody. The absence of immunostaining confirms the specificity of the signal. Actin was counterstained with Rhodamine-Phalloidin (red), and nuclei were counterstained with Dapi (blue). The counterstain controls distinguish major anatomical features such as the mouth, excretory pore, excretory cell, inner body, anus, and tail.
