# Supplementary material for: Genetic polymorphism, constitutive expression and tissue localization of Dirofilaria immitis P-glycoprotein 11: a putative marker of macrocyclic lactone resistance
Source: Parasit Vectors. 2022 Dec 21;15:482. doi: 10.1186/s13071-022-05571-6 (PMC9773537; doi:10.1186/s13071-022-05571-6)
Supplement: Supplementary file 7 — Additional file 7: Figure S5. Raw thermogradient (51–63 °C) constitutive expression data of: a Dirofilaria immitis Pgp-11, b D. immtis GAPDH, c D. immitis Actin, d D. immitis pmp-3, e D. immitis Histone H3, in the Missouri isolate, determined using droplet digital PCR [file 13071_2022_5571_MOESM7_ESM.docx]

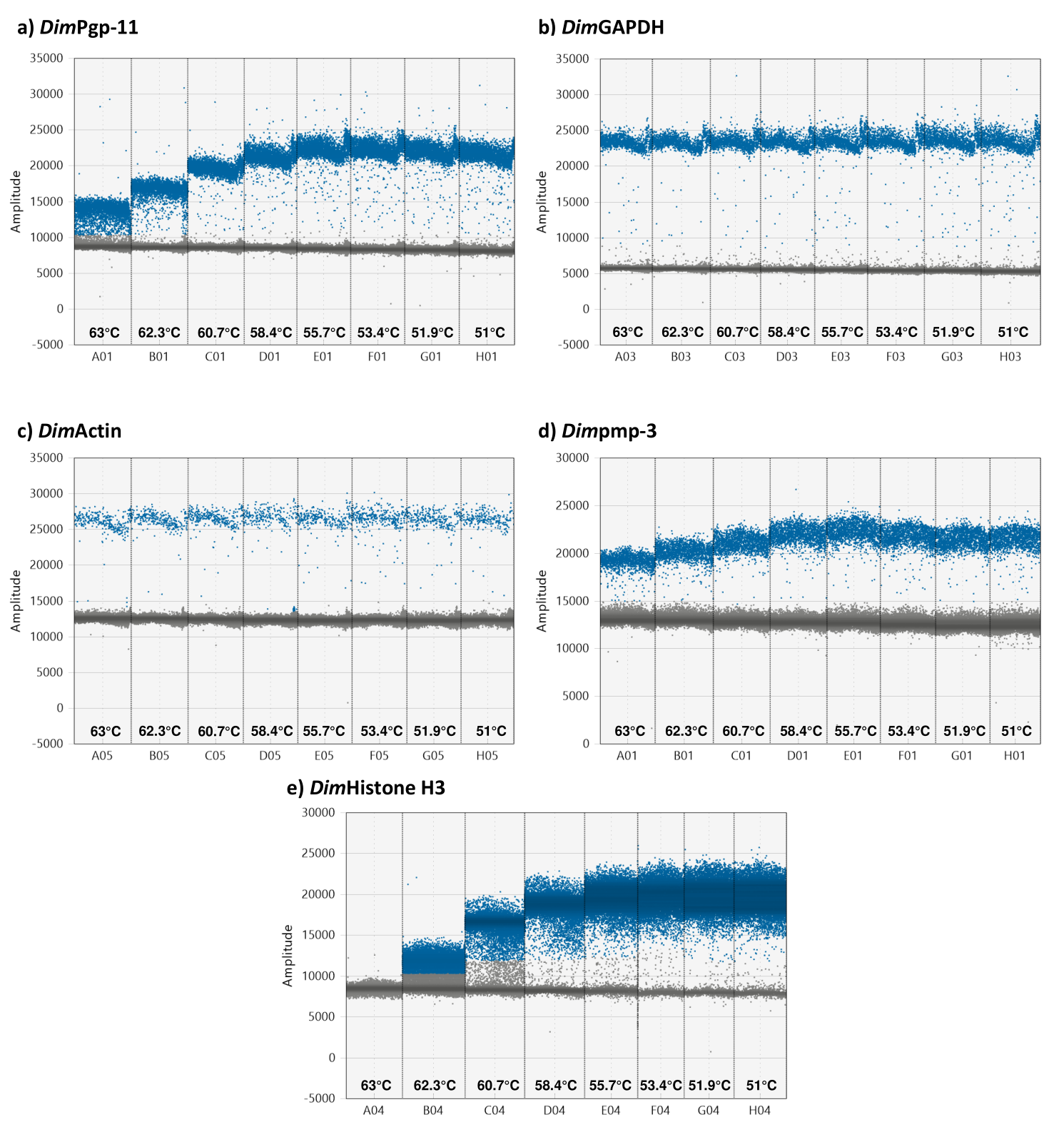


**Figure 3** Raw thermogradient (51 ° C – 63 ° C) transcript level data of a) *Dirofilaria immitis* Pgp-11 and b) *D. immtis* GAPDH c) *D. immitis* Actin d) *D. immitis* pmp-3 e) *D. immitis* Histone H3 in the Missouri isolate determined using droplet digital PCR.
